# Boolean implication analysis of single-cell data predicts retinal cell type markers

**DOI:** 10.1186/s12859-022-04915-4

**Published:** 2022-09-16

**Authors:** Rohan Subramanian, Debashis Sahoo

**Affiliations:** 1 Harvey Mudd College, Claremont, CA USA; 2 Department of Pediatrics, University of California San Diego, 9500 Gilman Drive, MC 0703, Leichtag Building 132, La Jolla, CA 92093-0831 USA; 3 Department of Computer Science and Engineering, Jacobs School of Engineering, University of California San Diego, La Jolla, CA USA

**Keywords:** Retina, Single-cell RNA sequencing, Pluripotent stem cells, Boolean analysis, Bioinformatics

## Abstract

**Background:**

The retina is a complex tissue containing multiple cell types that are essential for vision. Understanding the gene expression patterns of various retinal cell types has potential applications in regenerative medicine. Retinal organoids (optic vesicles) derived from pluripotent stem cells have begun to yield insights into the transcriptomics of developing retinal cell types in humans through single cell RNA-sequencing studies. Previous methods of gene reporting have relied upon techniques in vivo using microarray data, or correlational and dimension reduction methods for analyzing single cell RNA-sequencing data computationally. We aimed to develop a state-of-the-art Boolean method that filtered out noise, could be applied to a wide variety of datasets and lent insight into gene expression over differentiation.

**Results:**

Here, we present a bioinformatic approach using Boolean implication to discover genes which are retinal cell type-specific or involved in retinal cell fate. We apply this approach to previously published retina and retinal organoid datasets and improve upon previously published correlational methods. Our method improves the prediction accuracy of marker genes of retinal cell types and discovers several new high confidence cone and rod-specific genes.

**Conclusions:**

The results of this study demonstrate the benefits of a Boolean approach that considers asymmetric relationships. We have shown a statistically significant improvement from correlational, symmetric methods in the prediction accuracy of retinal cell-type specific genes.

Furthermore, our method contains no cell or tissue-specific tuning and hence could impact other areas of gene expression analyses in cancer and other human diseases.

**Supplementary Information:**

The online version contains supplementary material available at 10.1186/s12859-022-04915-4.

## Introduction

Characterization of retinal cell types is an important field of study with wide applications in ophthalmology and regenerative medicine. With the advent of single cell RNA-sequencing (scRNA-seq), computational methods for gene reporting can yield valuable insights into genes that are important in determining cell fate [1]. Human pluripotent stem cells (hPSCs) can be used to generate retinal cell types in vitro with potential applications to cure age-related macular degeneration, retinitis pigmentosa and other retina-related causes of blindness. However, gene reporting and characterization of these cell types is difficult as they differentiate asynchronously in complex cultures [2]. In addition, more datasets of mouse models exist compared to human or organoid models. We propose using Boolean implication analysis to improve the prediction accuracy of existing correlational methods for gene reporting.

### Previous methods in vivo and in vitro

One of the most common methods to study the effect of key genes on retinal development is the use of genetically modified “knockout” murine models, which are frequently used to validate differentially expressed genes from microarray data [3–20]. Fluorescent gene reporter lines are widely used to check for gene expression in single cells, or purified populations of a single cell type [2, 21–25]. Bulk RNA sequencing (RNA-seq) has helped define the transcriptomes of larger populations of retinal cell types [3, 9, 14, 17, 21, 24, 26–35]. To study the characteristics of isolated cells or droplets, flow cytometry was formerly a major method [36, 37]. Single-cell RNA sequencing (scRNA-seq) is increasingly common today and is one the most detailed methods to profile transcriptomes of retinal cell types and subtypes [2, 8, 13, 22, 38–48].

Most studies on retinal cell types have relied upon murine models, but many increasingly study human donor retinas [6, 30, 31, 48–50], especially in order to profile retinal disease [31, 43, 50–53]. Glaucoma, age-related macular degeneration and retinal light damage have also been studied in murine models [7, 14, 29, 34, 35, 54, 55]. Some studies have grown cell lines in vitro from fetal retina [49, 56], whereas others have used human pluripotent, induced pluripotent or embryonic stem cells to generate purified cell populations or retinal organoids [2, 3, 8, 28, 38, 57–59]. In order to study the development of retinal cell types over time, the lineage of stem cell progeny [58] and time course data from different time points (using PCR and RNA-seq) have been investigated [39, 41, 54].

### Previous computational methods

Differential expression analysis is the most common method to identify retinal cell type-specific genes and biomarkers from microarray, RNA-seq and scRNA-seq data [10, 13, 14, 17, 24, 29–31, 39, 41, 46, 47, 53, 56, 59]. In single-cell analysis, dimension reduction through Principal Component Analysis to reduce the size of data and allow visualization is often performed before hierarchical clustering identify cell clusters [2, 7, 30, 41, 42, 49, 56, 60]. Cell clusters can be assigned to different cell types or subtypes based on the expression of key marker genes [48]. AI-guided identification of cell clusters has recently been investigated [61].

scRNA-seq data provides opportunities for in-depth analysis of the transcriptome of individual cells, and subsequent characterization of cell types, subtypes and regions of retina. However, scRNA-seq data is highly noisy, and contains large numbers of zeroes, among which true and false negatives are indistinguishable. Many of these zeroes are dropouts, caused by a failure to capture or amplify a transcript. As a result, scRNA-seq data generates sparse arrays with low false omission rate and high negative predictive values [62].

Most studies, to date, have been highly dependent on cell clustering, which is not always achievable, especially in datasets containing immature or developing cells [1]. Pseudo-time analysis, which maps single-cell trajectories along developmental processes, has been applied to retinal organoids, and takes into account transitory states rather than discrete clusters [38]. However, these approaches are hindered by asynchronous differentiation of cell types in retina and the symmetric nature of clustering algorithms [63]. Correlational methods for ranking gene expression are also widely used, bypassing the need to discover cell clusters and identifying co-expressed genes in complex cultures, including developing retinal organoids [2, 8, 23, 27, 49, 64].

Identifying relationships between genes has led towards broader goals of graph [47, 60] and network-based analysis [9, 10, 17, 25, 27, 31, 60, 65]. Gene expression networks can be used to identify transitions between phenotypes and disease states, paving the way for clinical target identification. Correlational analysis is traditionally used to derive co-expression networks, and knockout murine models are used to directly investigate the effect of one gene’s absence. However, the symmetric nature of correlation can lead to loss of valuable information and does not provide insight into the expression of genes over time. Bayesian networks of gene regulation and expression in the retina mainly identify transcription factors and their targets [60, 66]. Hence, the motivation of our work was to develop a universally applicable state-of-the-art method that filtered out noise, could be applied to a wide variety of datasets and lent insight into gene expression over differentiation.

### A Boolean approach

Boolean logic is a simple mathematical relationship between two values such as high/low or 1/0. We propose using Boolean implication (“if–then” relationships) to study the dependency between genes from scRNA-seq data. Research by Sahoo et al. has shown that analysis of Boolean implication relationships is better at filtering out noise than a correlational approach [67]. Analysis of Boolean implication lends insight into asymmetric relationships disregarded by correlation.

While Boolean implication, like correlation, does not imply causation, asymmetric Boolean relationships can be thought of in terms of subsets. For example, the relationship Gene A high ⇒ Gene B high indicates that all cells with Gene B high are a subset of those with Gene A high. This allows for analysis of developmentally regulated genes using Boolean implication, first pioneered in the MiDReG tool published by Sahoo et al. [68].

In previous research, Boolean methods have led to the discovery of prognostic biomarkers for bladder and colon cancer [69–71]. These methods have also led to characterization of hematopoietic stem cells and identification of B and T cell precursors [72, 73]. Our methods have not previously been applied to stem cell-derived retinal cell types, but have yielded insights into changes in transcriptional profiles of healthy retina and retinoblastoma [74]. The StepMiner and BooleanNet algorithms were developed for microarray data by Sahoo et al. to identify Boolean implication relationships between genes, but have since been applied to a wide variety of high-throughput data, such as RNA-seq, scRNA-seq and microbiome data [68, 75–77].

A small number of previous studies have used single-cell RNA-seq data to construct gene regulatory networks that use Boolean relationships such as AND, OR and NOT to model processes such as hematopoiesis [78]. These studies also begin with binarizing the data to build dynamic executable models (sequential logic with memory) that are classically different from Boolean implication relationships which follows combinational logic (memoryless). Qiu 2020 recognizes binarizing gene expression values can “embrace the dropouts” in single-cell data by using zero values in the data to characterize cell types [79]. However, the data is binarized by simply replacing any non-zero values with 1, losing the quantitative information of gene expression. In this work, the StepMiner algorithm computes a threshold that considers the quantitative expression values before binarizing them as low or high. This approach focuses on Boolean implication relationships as they can identify cell populations based on a relationship between two genes and shed light on gene expression during differentiation.

## Methods

### Data normalization and annotation

We applied log_2_(v + 1) transformation to TPM values from the Phillips 2018 dataset (GSE98556, n = 546) and the Macosko 2015 dataset (GSE63472, n = 49,300). Log-transformed CPM values were used for analysis of GSE84859 (n = 14), GSE98838 (n = 22), GSE130636 (n = 8217), GSE148077 (n = 86,253) and the Lu 2020 dataset (GSE138002 GSE11606 GSE122970, n = 118,555).

Cells were annotated with clinical characteristics, and data were uploaded to hegemon.ucsd.edu/eye where they are publicly available. In the Hegemon online tool, scatter plots between genes are generated, with each point representing the expression level of the genes in a single cell [67–71].

### Discovering Boolean implications

#### StepMiner algorithm

The StepMiner algorithm identifies thresholds to convert continuous expression values into discrete values by fitting a step function to sorted values. A step can be defined as the sharpest increase in sorted gene expression values over an interval. Having identified a threshold *t*, gene expression values greater than *t* + 0.5 are considered high, and those below *t* − 0.5 are considered low. Those between *t* + 0.5 and *t* − 0.5 are considered intermediate, where 0.5 is a margin of error equivalent to more than a two-fold change from high to low values. Points in the intermediate region are ignored while determining the type of Boolean relationship, as they are likely to appear on the wrong side of the threshold due to random error. These thresholds are used to divide the plot into four quadrants (see Additional file 1: Fig. S1G) [67, 80].

#### BooleanNet algorithm

The BooleanNet algorithm identifies the type of Boolean implication relationship by identifying the sparse quadrant(s) using a statistic *S* and likelihood error rate *p*. There are six types of Boolean implication relationship: high ⇒ high, low ⇒ low, high ⇒ low, low ⇒ high, equivalent and opposite. The first four are asymmetric and have only one sparse quadrant. The latter two are symmetric and have two sparse quadrants. Further information can be found in Additional file 1: Fig. S1 [67, 80].

#### False discovery rate

For each scRNA-seq dataset we analyzed, we computed the false discovery rate (FDR) to evaluate the significance of Boolean implication relationships found. We randomly permuted the counts for each microbe independently 5 times and identified all Boolean relationships in this randomized dataset using the method described above [81]. The FDR is the ratio of the average number of Boolean relationships in the randomized dataset to the original dataset.

### Boolean approach to gene reporting of retinal cell types

**Fig. 1:**
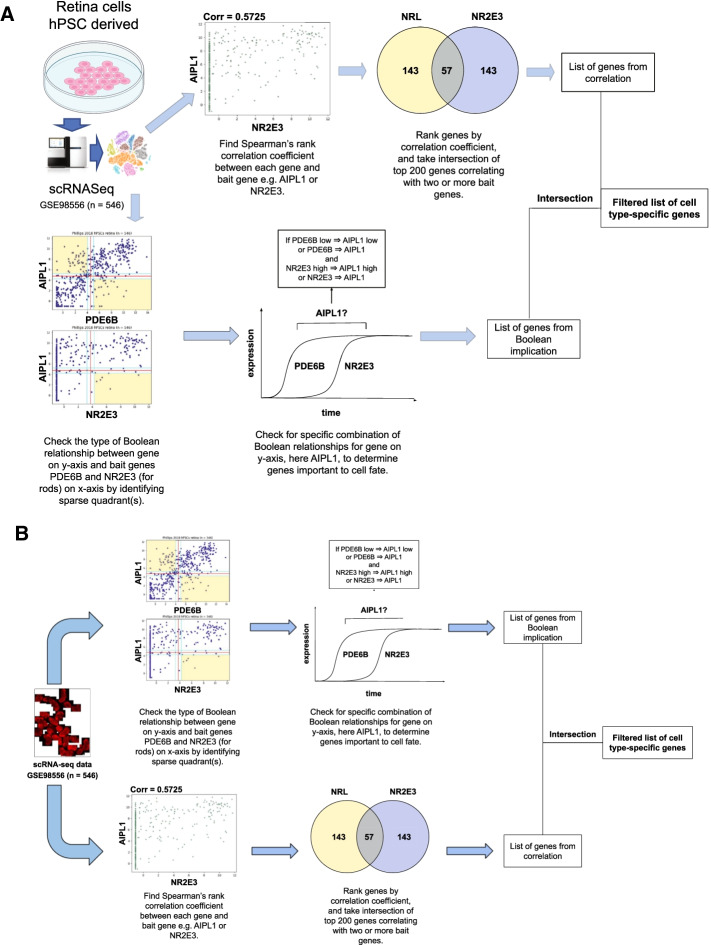
Schematic algorithm. Schematic algorithm to discover cell type-specific genes from scRNA-seq data by combining correlational and Boolean implication analysis. Boolean implication analysis uses one general and one specific bait gene to identify cell type-specific biomarkers. Spearman’s rank correlation coefficient analysis (SRCCA) uses one or more genes specific to a cell type as bait genes to identify other genes expressed in the same cell type. Boolean analysis is directly compared to SRCCA and improvement is tested using two proportion Z-test

We propose using the method described in Fig. 1 to identify specific markers of retinal cell types from single-cell datasets such as GSE98556 (n = 546) and GSE63472 (n = 49,300). We require two or more known genes for each cell type called “bait genes”. We searched for genes which had a low ⇒ low or equivalent Boolean relationship with the first bait gene and high ⇒ high or equivalent Boolean relationship with the second bait gene.

This specific combination of Boolean relationships is akin to searching for genes which have an impact on cell fate. If a gene passes this analysis, the set of cells where Gene X is low is a subset of the cells where the first bait gene is low, and the set of cells where Gene X is high is a subset of the cells where the second bait gene is high. This method can allow us to infer genes which are expressed after the first bait gene, and before the second bait gene during development. Hence, the choice of bait genes plays an important role in determining the results. We chose bait genes which led to shorter gene lists compared to correlational analysis, so that Boolean analysis could be used to filter correlational results. We also chose bait genes that yielded a greater number of known markers of these four retinal cell types. These were selected and verified from previous literature on rod and cone photoreceptors [6, 82, 83], retinal ganglion cells (RGCs) [23, 24] and retinal pigment epithelium (RPE) [84–88]. These genes are displayed in Additional file 3: Table S2, where they can be compared with existing databases such as CellMarker [83] and our results.

More than two bait genes can be considered by searching for high ⇒ high, low ⇒ low or equivalent Boolean relationships in two out of three bait genes instead of one out of two. This allows for combination of multiple cell type-specific marker genes in the analysis.

We discovered 35,389,605 Boolean implication relationships in GSE98556 with an FDR of 0.000958. We found 3,772,614 relationships in GSE63472 with an FDR of 0.0466. The distribution of the six types of Boolean implication relationships is displayed in a histogram in Additional file 1: Fig. S1K. In datasets used for this study, the four most common types of relationships are high ⇒ low, low ⇒ low, high ⇒ high, and equivalent. Opposite and low ⇒ high relationships are much rarer in the data and hence not considered. High ⇒ low relationships between genes are not useful for our purpose, since those genes are likely not expressed highly at the same time and may be expressed transiently during development. Hence, we focus on low ⇒ low, high ⇒ high, and equivalent relationships as they can identify marker genes that remain highly expressed together in a particular cell type.

### Spearman’s rank correlation coefficient

Spearman’s rank correlation coefficient (SRCC) is a nonparametric measure of the association between two ranked variables. We reviewed and reproduced the approach of Phillips et al. 2018, called Spearman’s rank correlation coefficient analysis (SRCCA). The correlation coefficient between bait genes and all other genes are found and ranked. Then, the intersection between the top 200 correlating genes with each bait gene is taken [2].

We combined both methods by taking the interaction of gene lists derived from both methods, hence filtering the list of correlating genes using Boolean implication as shown in Fig. 1. All analysis was performed using the Hegemon website, in Python 3 using the HegemonUtil and ScanPy libraries, and in R version 4.0.1.

### Thresholds for analysis

To account for noise in scRNA-seq data, thresholds for S and p are applied to adjust the sensitivity of the BooleanNet algorithm. To determine the most appropriate thresholds, we considered the false discovery rate (FDR) and the number of genes obtained. In previous work, S > 3 and *p* < 0.1 are generally considered for microarray data, where FDR < 0.001 is preferred [67]. However, single-cell data has more noise than microarray data due to dropouts, and S > 3 and *p* < 0.1 may not yield any Boolean relationships. Decreasing the S threshold relaxes the thresholds as a quadrant can still be considered sparse with a greater number of points in it. Increasing the p threshold also relaxes the thresholds by increasing the likelihood error rate. Relaxing the thresholds of the algorithm increases the number of Boolean implication relationships discovered but also increases the FDR.

We chose thresholds that led to an FDR less than 0.001 and a shorter, non-empty list of genes than SRCCA. Hence, thresholds for single-cell data were determined to be S > 2.5 and *p* < 0.35. While the FDR in GSE63472 is greater than 0.001, we used these thresholds to obtain a significant number of genes, which we then validated. To ensure that the list of genes from Boolean analysis was shorter than that from SRCCA, we increased p to 0.25 for rods in GSE98556.

### Quantification of results

Results were independently validated through differential expression. We evaluated whether genes were differentially expressed between rods and cones, and between photoreceptors and non-photoreceptor retinal cell types.

We selected and processed several validation datasets. Two were bulk RNA-seq datasets containing purified retinal cell types from Mus musculus: Hartl 2017 (GSE84589, n = 14) and Sarin 2018 (GSE98838, n = 22) [40, 46]. The third was a similar human retina scRNA-seq dataset, Voigt 2020 (GSE130636, n = 20,797) [48].

Using validation datasets with purified cell types, we checked for differential expression between retinal cell types by performing a one-tailed Welch’s t-test between the groups of cells to determine whether there was a statistically significant difference between the means of the two groups. Using this method, we could evaluate the proportion of genes which were specific to the cell type in question, expressed equally throughout the retina, and expressed in a different, non-target cell type.

Violin plots were generated from log-normalized CPM values using ScanPy [89]. Pseudobulk analysis in Additional file 1: Fig. S2 was performed by aggregating measurements in each cluster by taking the sum of raw counts for each cell type. CPM normalization, excluding highly expressed genes, was then performed to approximate the expression levels in each cell type.

## Results

### Boolean implication enables identification of cell type specific genes like SRCCA

Boolean implication analysis explores both symmetric and asymmetric relationships between genes whereas SRCCA only focuses on symmetric relationships. We hypothesize that application of asymmetric Boolean implication relationships may improve the accuracy of cell type-specific gene identification (Fig. 1).

**Fig. 2:**
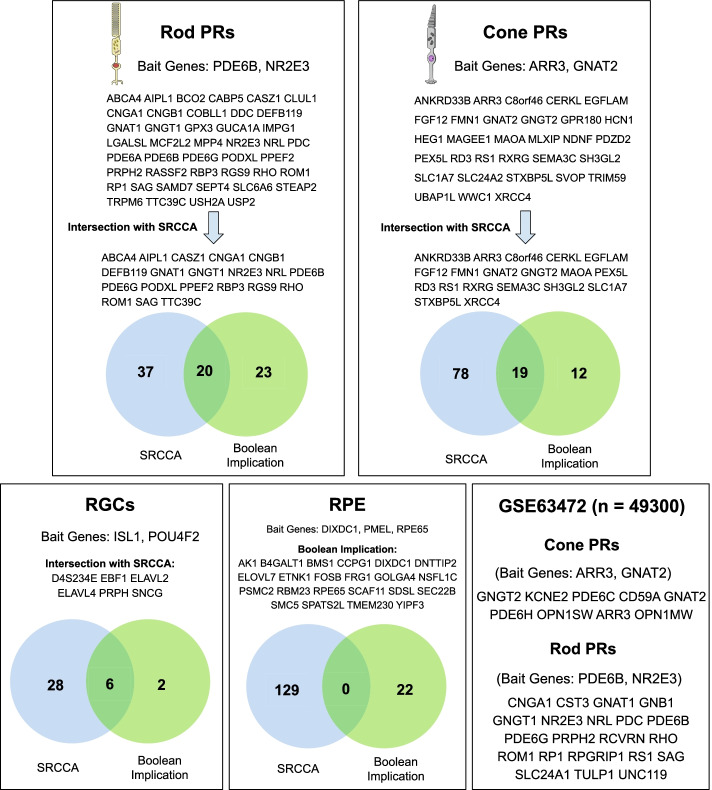
Results. Results of SRCCA and Boolean implication analysis of Phillips 2018 scRNA-seq dataset GSE98556 (n = 546) using two or more bait genes, for 5 retinal cell types. The bait genes for analysis are displayed at the top. For rod and cone PRs, genes from Boolean implication analysis are displayed, followed by intersection with SRCCA. For RGCs, only the genes from intersection of Boolean implication and SRCCA are displayed. For RPE, genes from Boolean implication are displayed as there are none from intersection. Results of Boolean implication analysis of the Macosko 2015 scRNA-seq dataset GSE63472 (n = 49,300) for 2 retinal cell types are also listed. Abbreviations: SRCCA, Spearman’s rank correlation coefficient analysis; PR, photoreceptors; RGC, retinal ganglion cell; RPE, retinal pigment epithelium

Application of Boolean implication analysis led to shorter lists of genes compared to SRCCA (Fig. 2). Selecting bait genes is crucial for both SRCCA and Boolean analysis. For Boolean analysis, a general marker and a more specific marker are ideal candidates. However, SRCCA relies only on specific bait genes. Because of these differences in specificity, we chose different set of bait genes for Boolean analysis from known marker genes for each retinal cell type. The same bait genes for rod and cone photoreceptors were used in the Boolean analysis of GSE98556 and GSE63472.

Application of Boolean analysis for gene reporting of photoreceptors led to longer lists of genes than other cell types. The largest intersection between SRCCA and Boolean implication was observed in rod photoreceptors. The gene lists derived from GSE63472 were shorter than those from GSE98556. The number of genes from Boolean implication in other retinal cell types such as RGCs and RPE was far lower than photoreceptors.

For RPE, three bait genes were chosen due to the excessively small number of genes obtained from two bait genes. This is likely to be due to the smaller number of cells from these types present in our dataset (GSE98556), compared to photoreceptors. The complete absence of intersection between genes from SRCCA and Boolean in RPE could also be explained by the very small number of RPE cells present in optic vesicle cultures produced by the method used by Phillips et al. [2]

### Filtering SRCCA using Boolean implication improves prediction accuracy

**Fig. 3:**
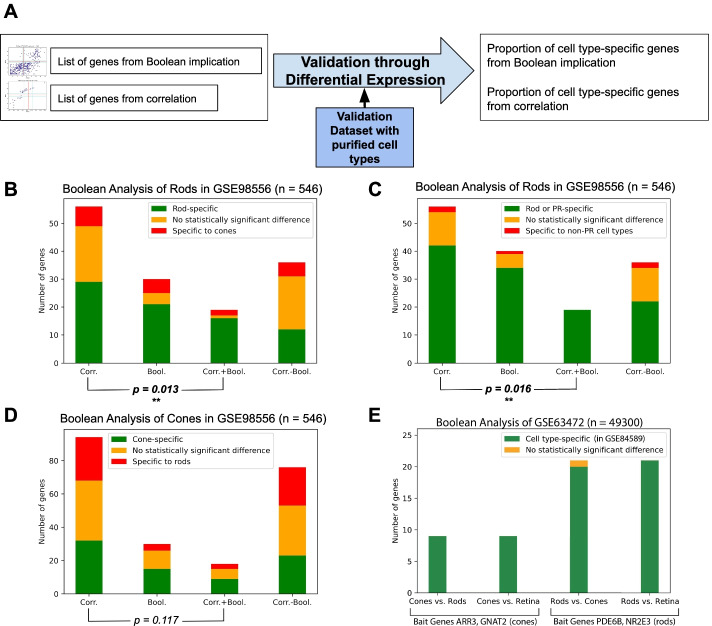
Independent validation of results. **A** Validation bulk RNA-seq datasets such as GSE84589 containing purified rods and cones from Mus musculus were used to validate rod and cone gene lists through differential expression. **B** Rod cell type-specificity of rod gene lists from 4 methods: Boolean implication, SRCCA, SRCCA filtered using Boolean implication and SRCCA without Boolean implication. **C** Photoreceptor-specificity of rod gene lists from 4 methods. **D** Cone cell type-specificity of rod gene lists from 4 methods. **E** Validation of cone and rod genes from Boolean analysis of large single-cell dataset GSE63472. Validation of the genes’ specificity was assessed in bulk dataset GSE84589. “Cones vs. Rods” denotes the specificity of putative cone genes in comparison with rods, and vice versa. “Vs. Retina” denotes the specificity of putative cone/rod gene in comparison with the entire retina. Abbreviations: Corr., correlation; Bool., Boolean; SRCCA, Spearman’s rank correlation coefficient analysis. *Note*: *P* values are from two-proportion Z-test between proportion of cell type-specific genes in lists from SRCCA and SRCCA filtered using Boolean implication

We independently validated the genes from SRCCA and Boolean implication using bulk RNA-seq datasets with purified retinal cell types (Fig. 3A). In Fig. 3B, there is a visible improvement in proportion of rod-specific genes while taking the intersection of SRCCA and Boolean implication. Similarly, the majority of SRCCA genes absent in Boolean implication were not specific to rods, or specific to cones. We were able to show a statistically significant improvement in the proportion of rod PR-specific genes by filtering correlating genes using Boolean implication. The proportion of genes rod-specific genes from SRCCA, 29 out of 56 (0.517), was improved to 16 out of 19 (0.842) by filtering using Boolean implication. This proportion was shown to be statistically significant by performing a two-proportion Z-test, returning a p-value of 0.013.

Similarly, as shown in Fig. 3C, we were able to show a statistically significant improvement in photoreceptor-specificity of the rod genes using the combined correlational and Boolean approach. All 19 genes obtained by filtering SRCCA using Boolean implication were photoreceptor-specific, and the p-value from the two-proportion Z-test was 0.016.

As seen in Fig. 3D, prediction accuracy of both SRCCA and Boolean analysis was lower in cone photoreceptors. The proportion of cone-specific genes, 15 out of 30 (0.500), was still highest in Boolean implication. Here, the prediction accuracy of Boolean methods alone was not improved by taking the intersection with SRCCA. However, this result could not be shown to be statistically significant due to the larger number of total genes in SRCCA. Hence, we evaluated the performance of Boolean analysis in a larger and more comprehensive dataset.

We also performed Boolean implication analysis for rod and cone photoreceptors using the same bait genes in GSE63472 (Macosko P14 mouse retina). Both GSE63472 and GSE98556 encompass the developmental stage of the retina, though GSE63472 comes from the Drop-seq of mouse retina. GSE63472 is more comprehensive due to its larger size (49,300 vs. 546 cells) and likely contains more identifiable retinal cell types than GSE98556 [42].

As seen in Fig. 3E, the list of 9 cone genes from GSE63472 was highly specific. All genes were enriched in cones compared to both rods and the whole retina. The list of 21 rod genes was also highly focused and only one gene showed no statistically significant difference in expression between cones and rods. The overall proportion of cell type-specific genes from Boolean analysis of GSE63472 was higher than those from GSE98556 (Fig. 3B–D).

### Boolean implication improves prediction accuracy of novel high confidence genes

Considering the overall improvement in prediction accuracy through Boolean implication analysis, we also investigated several specific examples of new discoveries through this method.

Novel high confidence genes are an important contribution of computational methods for gene reporting. Identification of high confidence markers of retinal cell types using SRCCA alone may be arbitrary, but we show that Boolean implication can lend greater insight into the cell type-specific genes.

**Fig. 4:**
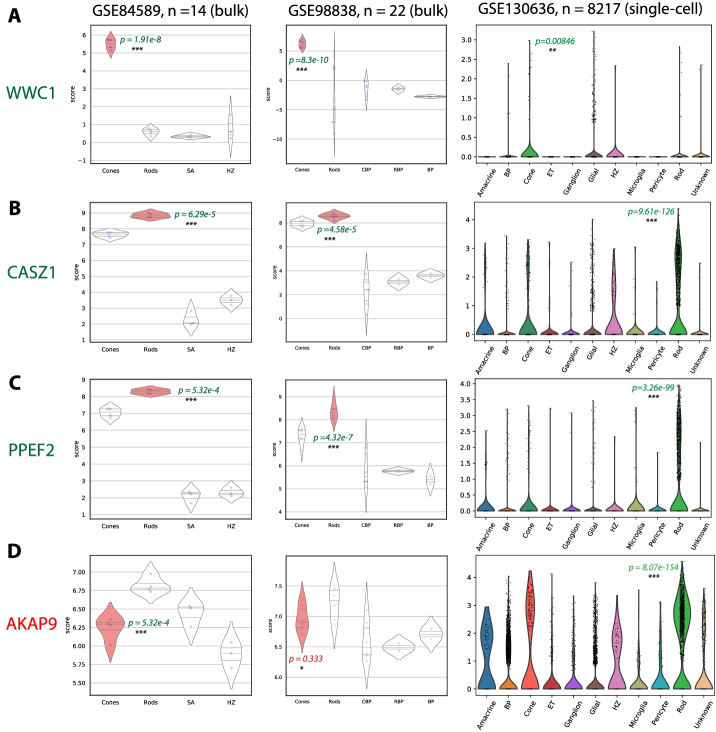
Specific examples. Further validation of high confidence photoreceptor genes in bulk, single-cell datasets. **A** Violin plots of WWC1 expression (putative cone-specific gene from Boolean analysis) in retinal cell types. **B**, **C** Violin plots of CASZ1 and PPEF2 expression (putative rod-specific genes from Boolean analysis) in retinal cell types. **D** Violin plots of AKAP9 expression (incorrect putative cone-specific gene from SRCCA) in retinal cell types. Abbreviations: SA, starburst amacrine cells; HZ, horizontal cells; CBP, cone bipolars; RBP, rod bipolars; BP, bipolars; ET, endothelial cells; SRCCA, Spearman’s rank correlation coefficient analysis. *Note*: *P* values from t-tests between highlighted cell type and all others

Boolean implication analysis identified WWC1 (WW domain containing protein-1) as a novel high confidence cone photoreceptor gene. This was validated independently in GSE84589 and GSE98838, with statistically significant overexpression in cone photoreceptors, as seen in the violin plots of Fig. 4A. In the human adult retina scRNA-seq dataset GSE130636 (n = 20,797), WWC1 is slightly enriched in cone photoreceptors but is also expressed in several other retinal cell types. This suggests that WWC1 may be involved in the cell fate of cones and a marker during early development. WWC1 has been described to have a broad function in the brain and memory by previous studies [90, 91].

Boolean implication analysis of rods also identified two novel rod-specific genes: CASZ1 (Castor zinc finger 1) (Fig. 4B) and PPEF2 (Protein Phosphatase with EF-Hand Domain 2) (Fig. 4C). These showed rod specificity in both validation datasets. CASZ1 is known to play a role in cell differentiation, and may hence play a significant role in influencing rod cell fate [92]. PPEF2 has been documented in rods before but has had several conflicting studies on its importance in rods [93, 94]. This documents its rod-specific function in human or hPSC-derived retina. Boolean implication analysis has shed light on potential novel markers of cone and rod photoreceptors.

In Additional file 1: Fig. S2, these genes were also validated in large and recent scRNA-seq datasets such as Peng 2020 (GSE148077, n = 86,253) [95] and Lu 2020 (GSE138002 GSE122970 GSE116106, n = 118,555) [41]. GSE148077 (Additional file 1: Fig. S2A) displayed rod-specific expression of PPEF2 and CASZ1. WWC1 is enriched in cones, but also shows expression in Muller Glia. In the Lu 2020 dataset, GSE138002 (Additional file 1: Fig. S2B) shows cone-specific expression of WWC1 and rod-specific expression of PPEF2 and CASZ1. CASZ1 also shows expression in retinal progenitor cells, suggesting that it continues to be expressed as progenitors develop into rods. GSE122970 (Additional file 1: Fig. S2C) shows that PPEF2 and CASZ1 are specifically expressed in rods. WWC1 appears to be expressed solely in rods, but this is likely due to the very small number (20) of cones in the dataset. Human embryonic cells in the Lu 2020 dataset (GSE116106) displayed different expression patterns, as they belong to a much earlier stage of development and differentiation is less advanced. GSE138002 contains only adult retinal cells and GSE122970 is comprised of human neonatal cells, and hence show differential expression of the genes identified.

Boolean implication analysis refuted AKAP9 (A-kinase anchoring protein-9), identified to be a high confidence cone photoreceptor gene by Phillips et al. 2018 based on the results from SRCCA. Figure 4D shows that it is not differentially expressed in cones and is more rod-specific as per GSE84589 and GSE130636.

## Discussion

Boolean methods improved upon correlational methods by filtering out noise and identifying asymmetric relationships that lend insight into the specificity of genes. Filtering correlating genes led to a statistically significant improvement in rod and photoreceptor-specificity for rod genes, and reproducibility for cone photoreceptor genes. Boolean analysis of GSE98556 allowed for comparison of Boolean and correlational methods and analysis of GSE63472 allowed us to show the efficacy of Boolean analysis in large single-cell datasets. Hence, we have shown that a combination of Boolean implication analysis and SRCCA improves the prediction accuracy of retinal cell type markers.

Boolean implication analysis provided more accurate insight into high confidence genes and led to the identification of WWC1 as a novel marker gene for cone photoreceptors. From literature, it is established that ARR3 is expressed > 60 days after the first appearance of cones during development [8]. The Boolean relationships ARR3 equivalent WWC1 and GNAT2 equivalent WWC1 allow us to conclude that these genes are expressed around a similar time and continue to stay high expressed later during cone development and mature cones. Previous attempts to identify high confidence genes from extensive gene lists obtained through SRCCA alone have no way to distinguish between noise and true cell type-specific genes. The asymmetric nature of Boolean relationships allows us to determine whether a gene is expressed more generally or specifically, which is not present in correlation.

Another advantage of Boolean implication is that the analysis can always be performed over the entire dataset. Boolean implication relationships between genes are best visible when there is a greater diversity of cell types, including those not expressing the gene. However, SRCCA generally requires the operator to choose a specific subset of the data (e.g. day 70) on which to perform the analysis, based on whether the cell type in question is present at that developmental stage. This choice has a significant effect on the result of SRCCA, and an inept choice of the subset may lead to false associations not generalizable over larger datasets. This issue can be solved using Boolean implication.

However, Boolean implication analysis was also not entirely free from error. The main source of error appears to be the dropouts, which lead to a greater density of points in quadrants a_10_, a_00_ and a_01_ in many cases. These artefactual zeroes may lead to incorrect interpretations of gene expression over time or during differentiation. Along with the slightly relaxed thresholds adapted for scRNA-seq, this increased the false discovery rate, especially in GSE63472. This issue likely reduced the improvement in quality of analysis in cone photoreceptors. Even so, a combination of correlational and Boolean implication analysis in GSE98556 could lead to completely error-free results in some cases (Fig. 3C). Boolean analysis in GSE63472 led to almost error-free results in cones and rods (Fig. 3E), likely due to the more comprehensive nature of the dataset. To filter out noise such as dropouts, we filtered our list and tuned the thresholds to yield fewer genes, especially in GSE63472, which may lead to false negatives. However, the aim of our approach is to provide a method that is more specific than sensitive. For the aim of this study, a small number of highly specific and conserved markers is more useful compared to other methods such as correlation and differential expression, which yield more genes but also fail to filter out noise.

There were differences in the performance of our methods between different cell types. In cell types present in smaller numbers in the retina, we can observe that the number of genes from Boolean analysis alone and combined with SRCCA is also smaller. The analysis performed best in rods, the most numerous neural retina cell type, followed by cones [96]. The number of genes obtained for RGCs was also lower, and some genes such as EBF1, ELAVL2 and ELAVL4 are also expressed in amacrine cells. Phillips et al. describe the results of their organoid generation protocol, which contained a lower proportion of RGCs compared to photoreceptors. They estimate that the number of RGCs declined over development [2]. In RPE, which is rarely present in the optic vesicle culture protocol employed by Phillips et al. 2018, there was no intersection between Boolean analysis and SRCCA, indicating that the results in that case may contain many false positives. In addition, RPE-associated genes such as ADH1A3 [97] and RLBP1 [98] are absent from the list, as these genes have no Boolean relationships with the bait genes in GSE98556. This is likely because the percentage of ADH1A3-high (3.6%) and RLBP1-high cells (7.3%) is very small and there are not enough values in the high-high quadrant. Comparing results across cell types, the quality of putative marker genes obtained is dependent on the comprehensiveness of the dataset. However, there is no link between the number of genes obtained from SRCCA and the population of cell type, as a fixed number of top correlating genes are considered. Hence, Boolean analysis can lend insight into the cell types for which the data is comprehensive enough to provide accurate resolution.

The method of independent validation considered several datasets to evaluate specificity and reproducibility. These high-quality bulk RNA-seq datasets provided reliable results for most genes, as many genes from our analysis of hPSC-derived retina are conserved across human and mouse retina. However, it was not infallible due to small variations between the species. The validation of gene lists from GSE63472 (Mus musculus) identified more specific genes than that of GSE98556 (human), which can be attributed to species variation in addition to the higher specificity of the analysis in GSE63472. Larger and more comprehensive single-cell datasets such as GSE130636, GSE148077 and the Lu 2020 dataset validated the genes in cell types from in vivo derived human retina. These shared cell type markers demonstrate the utility of stem cell-derived organoids to emulate in vivo samples. Even so, the results for some cell types such as RGCs and RPE were not as specific when validated using in vivo derived retina datasets. In vivo derived retina contains several million RPE and this number is proportional to the number of rods and cones. However, this is clearly not the case in GSE98556 (hPSC-derived retinal organoid) [99]. In this regard, our analysis highlights differences between cell populations in hPSC and in vivo derived retina and how it impacted results.

Boolean implication analysis provides all the advantages offered previously by SRCCA including efficiency, ability to combine multiple bait genes, and improved prediction accuracy compared to earlier methods. Our method can allow researchers to analyze single-cell data even when cell clusters cannot be identified, a common issue in datasets containing developing cells. Combining both methods provides statistically significant improvements in specificity and reproducibility of genes. Boolean implication can be easily inferred from scatter plots on the Hegemon online tool, making it an intuitive option for biologists and computer scientists alike [76].

## Conclusion

In this work, we have developed a novel approach for analysis of scRNA-seq data based on Boolean implication. We have shown a statistically significant improvement in the prediction accuracy of retinal cell-type specific genes, as compared to earlier approaches based solely on correlation. Application of our method to retinal organoid datasets identified novel high confidence cell type-specific genes such as WWC1 for cones and CASZ1 and PPEF2 for rods. This Boolean approach allows for analysis and characterization of cell types in complex cultures, even when cell clustering cannot be achieved. Considering asymmetric relationships has allowed us to effectively filter out noise, lending insight into genes with potential importance in regenerative medicine.

## Supplementary Information

**Additional file 1. Figure S1:** Method for discovering and applying Boolean implication relationships in single cell RNA sequencing data. (A-F): Six types of Boolean implication relationships are visible on scatterplots. Two are symmetric with two sparse quadrants (A,B) and four are asymmetric with one sparse quadrant (C-F). (G): This plot is divided into four quadrants based on thresholds identified by the StepMiner algorithm. (H-I): The BooleanNet algorithm identifies the sparse quadrants using a statistic S and a likelihood error rate p and applying thresholds of 2.5 and 0.35, respectively. (J): Analysis of Boolean implication relationships was used to find genes involved in cell fate determination using bait genes (A and B). genes (A and B). (K): Distribution of the six types of Boolean implication relationship (as seen in A-F) in the single-cell dataset GSE98556. The log-log plot shows a histogram of the number of each relationship in GSE98556 and the number of genes exhibiting that relationship.**Figure S2**. Single-cell validation (A): Violin plots in the Peng 2020 dataset (GSE148077). (B): Violin plots in adult retinal cells of the Lu 2020 dataset (GSE138002). (C): Violin plots in human neonatal retina of the Lu 2020 dataset (GSE122970). Violin plots (left x-axis) are generated from log-normalized CPM values. Line graphs (right x-axis) represent pseudobulk expression values for each cell type, normalized using CPM. WWC1 is a proposed cone photoreceptor gene, and CASZ1 and PPEF2 are proposed rod photoreceptor genes.

**Additional file 2.** High confidence markers in single-cell validation datasets.

**Additional file 3.** Comparison of Boolean implication analysis with known markers of retinal cell types.

## Data Availability

All data is available in public repository and the relevant accession numbers are provided in the text and the supplementary materials. Datasets GSE98556 and GSE63472 were analyzed and GSE84859, GSE98838, GSE130636, GSE148077, GSE138002 and GSE122970 were used for validation. Datasets can be queried at hegemon.ucsd.edu/eye. To reproduce Hegemon scatter plots between genes, click “Explore” after entering gene names, and then download as a high-resolution PDF by clicking “Download”. Code to reproduce all other figures is available in a Jupyter notebook at https://github.com/RohanS14/Boolean-lab/blob/main/Boolean-Retina-Analysis-Revised.ipynb.

## References

[1] Zerti D (2020). Understanding the complexity of retina and pluripotent stem cell derived retinal organoids with single cell RNA sequencing: current progress, remaining challenges and future prospective. Curr Eye Res.

[2] Phillips MJ (2018). A novel approach to single cell RNA-sequence analysis facilitates in silico gene reporting of human pluripotent stem cell-derived retinal cell types. Stem Cells.

[3] Brooks MJ (2019). Improved retinal organoid differentiation by modulating signaling pathways revealed by comparative transcriptome analyses with development in vivo. Stem Cell Rep.

[4] Brooks MJ (2011). Next-generation sequencing facilitates quantitative analysis of wild-type and Nrl(-/-) retinal transcriptomes. Mol Vis.

[5] Cheng H (2006). In vivo function of the orphan nuclear receptor NR2E3 in establishing photoreceptor identity during mammalian retinal development. Hum Mol Genet.

[6] Corbo JC (2007). A typology of photoreceptor gene expression patterns in the mouse. Proc Natl Acad Sci USA.

[7] Howell GR (2011). Molecular clustering identifies complement and endothelin induction as early events in a mouse model of glaucoma. J Clin Invest.

[8] Kallman A (2020). Investigating cone photoreceptor development using patient-derived NRL null retinal organoids. Commun Biol.

[9] Kim JW (2016). NRL-regulated transcriptome dynamics of developing rod photoreceptors. Cell Rep.

[10] Ma H (2013). Loss of cone cyclic nucleotide-gated channel leads to alterations in light response modulating system and cellular stress response pathways: a gene expression profiling study. Hum Mol Genet.

[11] Mizeracka K, DeMaso CR, Cepko CL (2013). Notch1 is required in newly postmitotic cells to inhibit the rod photoreceptor fate. Development.

[12] Montana CL (2013). Reprogramming of adult rod photoreceptors prevents retinal degeneration. Proc Natl Acad Sci USA.

[13] Mustafi D (2016). Transcriptome analysis reveals rod/cone photoreceptor specific signatures across mammalian retinas. Hum Mol Genet.

[14] Mustafi D (2011). Defective photoreceptor phagocytosis in a mouse model of enhanced S-cone syndrome causes progressive retinal degeneration. FASEB J.

[15] Onishi A (2010). The orphan nuclear hormone receptor ERRbeta controls rod photoreceptor survival. Proc Natl Acad Sci USA.

[16] Palczewska G (2016). Receptor MER tyrosine kinase proto-oncogene (MERTK) is not required for transfer of bis-retinoids to the retinal pigmented epithelium. J Biol Chem.

[17] Perez-Cervantes C (2020). Enhancer transcription identifies cis-regulatory elements for photoreceptor cell types. Development.

[18] Roger JE (2012). Preservation of cone photoreceptors after a rapid yet transient degeneration and remodeling in cone-only Nrl-/- mouse retina. J Neurosci.

[19] Sundermeier TR (2014). DICER1 is essential for survival of postmitotic rod photoreceptor cells in mice. FASEB J.

[20] Yoshida S (2004). Expression profiling of the developing and mature Nrl-/- mouse retina: identification of retinal disease candidates and transcriptional regulatory targets of Nrl. Hum Mol Genet.

[21] Buenaventura DF, Corseri A, Emerson MM (2019). Identification of genes with enriched expression in early developing mouse cone photoreceptors. Invest Ophthalmol Vis Sci.

[22] Cherry TJ (2009). Development and diversification of retinal amacrine interneurons at single cell resolution. Proc Natl Acad Sci USA.

[23] Langer KB (2018). Retinal ganglion cell diversity and subtype specification from human pluripotent stem cells. Stem Cell Rep.

[24] Sajgo S (2017). Molecular codes for cell type specification in Brn3 retinal ganglion cells. Proc Natl Acad Sci USA.

[25] Siegert S (2012). Transcriptional code and disease map for adult retinal cell types. Nat Neurosci.

[26] Cherry TJ (2020). Mapping the cis-regulatory architecture of the human retina reveals noncoding genetic variation in disease. Proc Natl Acad Sci USA.

[27] Dorrell MI (2004). Global gene expression analysis of the developing postnatal mouse retina. Invest Ophthalmol Vis Sci.

[28] Gill KP (2016). Enriched retinal ganglion cells derived from human embryonic stem cells. Sci Rep.

[29] Harder JM (2018). Jnk2 deficiency increases the rate of glaucomatous neurodegeneration in ocular hypertensive DBA/2J mice. Cell Death Dis.

[30] Li M (2014). Comprehensive analysis of gene expression in human retina and supporting tissues. Hum Mol Genet.

[31] Newman AM (2012). Systems-level analysis of age-related macular degeneration reveals global biomarkers and phenotype-specific functional networks. Genome Med.

[32] Ratnapriya R (2019). Retinal transcriptome and eQTL analyses identify genes associated with age-related macular degeneration. Nat Genet.

[33] Sugino K (2019). Mapping the transcriptional diversity of genetically and anatomically defined cell populations in the mouse brain. Elife.

[34] Williams PA (2017). Nicotinamide and WLD(S) act together to prevent neurodegeneration in glaucoma. Front Neurosci.

[35] Williams PA (2017). Vitamin B3 modulates mitochondrial vulnerability and prevents glaucoma in aged mice. Science.

[36] Carter DA, Dick AD, Mayer EJ (2009). CD133+ adult human retinal cells remain undifferentiated in Leukaemia Inhibitory Factor (LIF). BMC Ophthalmol.

[37] Portillo JA (2009). Identification of primary retinal cells and ex vivo detection of proinflammatory molecules using flow cytometry. Mol Vis.

[38] Collin J (2019). Deconstructing retinal organoids: single cell RNA-Seq reveals the cellular components of human pluripotent stem cell-derived retina. Stem Cells.

[39] Daum JM (2017). The formation of the light-sensing compartment of cone photoreceptors coincides with a transcriptional switch. Elife.

[40] Hartl D (2017). Cis-regulatory landscapes of four cell types of the retina. Nucleic Acids Res.

[41] Lu Y (2020). Single-cell analysis of human retina identifies evolutionarily conserved and species-specific mechanisms controlling development. Dev Cell.

[42] Macosko EZ (2015). Highly parallel genome-wide expression profiling of individual cells using nanoliter droplets. Cell.

[43] Orozco LD (2020). Integration of eQTL and a single-cell atlas in the human eye identifies causal genes for age-related macular degeneration. Cell Rep.

[44] Rheaume BA (2018). Single cell transcriptome profiling of retinal ganglion cells identifies cellular subtypes. Nat Commun.

[45] Roesch K, Stadler MB, Cepko CL (2012). Gene expression changes within Müller glial cells in retinitis pigmentosa. Mol Vis.

[46] Sarin S (2018). Role for Wnt signaling in retinal neuropil development: analysis via RNA-Seq and in vivo somatic CRISPR mutagenesis. Neuron.

[47] Shekhar K (2016). Comprehensive classification of retinal bipolar neurons by single-cell transcriptomics. Cell.

[48] Voigt AP (2019). Molecular characterization of foveal versus peripheral human retina by single-cell RNA sequencing. Exp Eye Res.

[49] Cui Z (2020). Transcriptomic analysis of the developmental similarities and differences between the native retina and retinal organoids. Invest Ophthalmol Vis Sci.

[50] Kirwan RP (2009). Differential global and extra-cellular matrix focused gene expression patterns between normal and glaucomatous human lamina cribrosa cells. Mol Vis.

[51] Bennis A (2015). Comparison of mouse and human retinal pigment epithelium gene expression profiles: potential implications for age-related macular degeneration. PLoS ONE.

[52] Charish J (2020). Neogenin neutralization prevents photoreceptor loss in inherited retinal degeneration. J Clin Investig.

[53] Galvao J (2018). The Kruppel-like factor gene target Dusp14 regulates axon growth and regeneration. Invest Ophthalmol Vis Sci.

[54] Agudo M (2008). Time course profiling of the retinal transcriptome after optic nerve transection and optic nerve crush. Mol Vis.

[55] Hadziahmetovic M (2012). Microarray analysis of murine retinal light damage reveals changes in iron regulatory, complement, and antioxidant genes in the neurosensory retina and isolated RPE. Invest Ophthalmol Vis Sci.

[56] Strunnikova NV (2010). Transcriptome analysis and molecular signature of human retinal pigment epithelium. Hum Mol Genet.

[57] Kuroda T (2017). Identification of a gene encoding slow skeletal muscle troponin T as a novel marker for immortalization of retinal pigment epithelial cells. Sci Rep.

[58] Hafler BP (2012). Transcription factor Olig2 defines subpopulations of retinal progenitor cells biased toward specific cell fates. Proc Natl Acad Sci USA.

[59] Chuang JH (2018). Expression profiling of cell-intrinsic regulators in the process of differentiation of human iPSCs into retinal lineages. Stem Cell Res Ther.

[60] Hu J (2010). Computational analysis of tissue-specific gene networks: application to murine retinal functional studies. Bioinformatics.

[61] Chen L (2020). Integrating deep supervised, self-supervised and unsupervised learning for single-cell RNA-seq clustering and annotation. Genes (Basel).

[62] Haque A (2017). A practical guide to single-cell RNA-sequencing for biomedical research and clinical applications. Genome Med.

[63] Saelens W (2019). A comparison of single-cell trajectory inference methods. Nat Biotechnol.

[64] Zhang SS (2006). A biphasic pattern of gene expression during mouse retina development. BMC Dev Biol.

[65] Howell GR (2011). Datgan, a reusable software system for facile interrogation and visualization of complex transcription profiling data. BMC Genomics.

[66] Qian J (2005). Identification of regulatory targets of tissue-specific transcription factors: application to retina-specific gene regulation. Nucleic Acids Res.

[67] Sahoo D (2008). Boolean implication networks derived from large scale, whole genome microarray datasets. Genome Biol.

[68] Sahoo D (2010). MiDReG: a method of mining developmentally regulated genes using Boolean implications. Proc Natl Acad Sci USA.

[69] Dalerba P (2011). Single-cell dissection of transcriptional heterogeneity in human colon tumors. Nat Biotechnol.

[70] Dalerba P (2016). CDX2 as a prognostic biomarker in stage II and stage III colon cancer. N Engl J Med.

[71] Volkmer JP (2012). Three differentiation states risk-stratify bladder cancer into distinct subtypes. Proc Natl Acad Sci USA.

[72] Inlay MA (2009). Ly6d marks the earliest stage of B-cell specification and identifies the branchpoint between B-cell and T-cell development. Genes Dev.

[73] Pang WW (2011). Human bone marrow hematopoietic stem cells are increased in frequency and myeloid-biased with age. Proc Natl Acad Sci USA.

[74] Rajasekaran S (2019). Non-coding and coding transcriptional profiles are significantly altered in pediatric retinoblastoma tumors. Front Oncol.

[75] Dabydeen SA, Desai A, Sahoo D (2019). Unbiased Boolean analysis of public gene expression data for cell cycle gene identification. Mol Biol Cell.

[76] Pandey S, Sahoo D (2019). Identification of gene expression logical invariants in Arabidopsis. Plant Direct.

[77] Vo D (2021). Boolean implication analysis unveils candidate universal relationships in microbiome data. BMC Bioinform.

[78] Schwab JD (2021). Reconstructing Boolean network ensembles from single-cell data for unraveling dynamics in the aging of human hematopoietic stem cells. Comput Struct Biotechnol J.

[79] Qiu P (2020). Embracing the dropouts in single-cell RNA-seq analysis. Nat Commun.

[80] Dang D (2020). Computational approach to identifying universal macrophage biomarkers. Front Physiol.

[81] Storey JD, Tibshirani R (2003). Statistical significance for genomewide studies. Proc Natl Acad Sci USA.

[82] de Melo J (2011). The Spalt family transcription factor Sall3 regulates the development of cone photoreceptors and retinal horizontal interneurons. Development.

[83] Zhang X (2019). Cell Marker: a manually curated resource of cell markers in human and mouse. Nucleic Acids Res.

[84] Bennis A (2017). Stem cell derived retinal pigment epithelium: the role of pigmentation as maturation marker and gene expression profile comparison with human endogenous retinal pigment epithelium. Stem Cell Rev Rep.

[85] Brandl C (2014). In-depth characterisation of Retinal Pigment Epithelium (RPE) cells derived from human induced pluripotent stem cells (hiPSC). Neuromol Med.

[86] Liao JL (2010). Molecular signature of primary retinal pigment epithelium and stem-cell-derived RPE cells. Hum Mol Genet.

[87] Plaza Reyes A (2020). Identification of cell surface markers and establishment of monolayer differentiation to retinal pigment epithelial cells. Nat Commun.

[88] Liu B (2019). Genetic analyses of human fetal retinal pigment epithelium gene expression suggest ocular disease mechanisms. Commun Biol.

[89] Wolf FA, Angerer P, Theis FJ (2018). SCANPY: large-scale single-cell gene expression data analysis. Genome Biol.

[90] Kremerskothen J (2003). Characterization of KIBRA, a novel WW domain-containing protein. Biochem Biophys Res Commun.

[91] Papassotiropoulos A (2006). Common Kibra alleles are associated with human memory performance. Science.

[92] Liu Z (2006). Molecular cloning and characterization of human Castor, a novel human gene upregulated during cell differentiation. Biochem Biophys Res Commun.

[93] Ramulu P (2001). Normal light response, photoreceptor integrity, and rhodopsin dephosphorylation in mice lacking both protein phosphatases with EF hands (PPEF-1 and PPEF-2). Mol Cell Biol.

[94] Sherman PM (1997). Identification and characterization of a conserved family of protein serine/threonine phosphatases homologous to Drosophila retinal degeneration C. Proc Natl Acad Sci USA.

[95] Yan W (2020). Cell atlas of the human fovea and peripheral retina. Sci Rep.

[96] Reese BE, Keeley PW (2016). Genomic control of neuronal demographics in the retina. Prog Retin Eye Res.

[97] Butler JM (2021). RNA-seq analysis of ageing human retinal pigment epithelium: Unexpected up-regulation of visual cycle gene transcription. J Cell Mol Med.

[98] Lidgerwood GE (2021). Transcriptomic profiling of human pluripotent stem cell-derived retinal pigment epithelium over time. Genomics Proteomics Bioinform.

[99] Panda-Jonas S, Jonas JB, Jakobczyk-Zmija M (1996). Retinal pigment epithelial cell count, distribution, and correlations in normal human eyes. Am J Ophthalmol.

