# Supplementary material for: Boolean implication analysis of single-cell data predicts retinal cell type markers
Source: BMC Bioinformatics. 2022 Sep 16;23:378. doi: 10.1186/s12859-022-04915-4 (PMC9482279; doi:10.1186/s12859-022-04915-4)
Supplement: Supplementary file 1 — Additional file 1. Figure S1: Method for discovering and applying Boolean implication relationships in single cell RNA sequencing data. (A-F): Six types of Boolean implication relationships are visible on scatterplots. Two are symmetric with two sparse quadrants (A,B) and four are asymmetric with one sparse quadrant (C-F). (G): This plot is divided into four quadrants based on thresholds identified by the StepMiner algorithm. (H-I): The BooleanNet algorithm identifies the sparse quadrants using a statistic S and a likelihood error rate p and applying thresholds of 2.5 and 0.35, respectively. (J): Analysis of Boolean implication relationships was used to find genes involved in cell fate determination using bait genes (A and B). genes (A and B). (K): Distribution of the six types of Boolean implication relationship (as seen in A-F) in the single-cell dataset GSE98556. The log-log plot shows a histogram of the number of each relationship in GSE98556 and the number of genes exhibiting that relationship.Figure S2. Single-cell validation (A): Violin plots in the Peng 2020 dataset (GSE148077). (B): Violin plots in adult retinal cells of the Lu 2020 dataset (GSE138002). (C): Violin plots in human neonatal retina of the Lu 2020 dataset (GSE122970). Violin plots (left x-axis) are generated from log-normalized CPM values. Line graphs (right x-axis) represent pseudobulk expression values for each cell type, normalized using CPM. WWC1 is a proposed cone photoreceptor gene, and CASZ1 and PPEF2 are proposed rod photoreceptor genes. [file 12859_2022_4915_MOESM1_ESM.pdf]

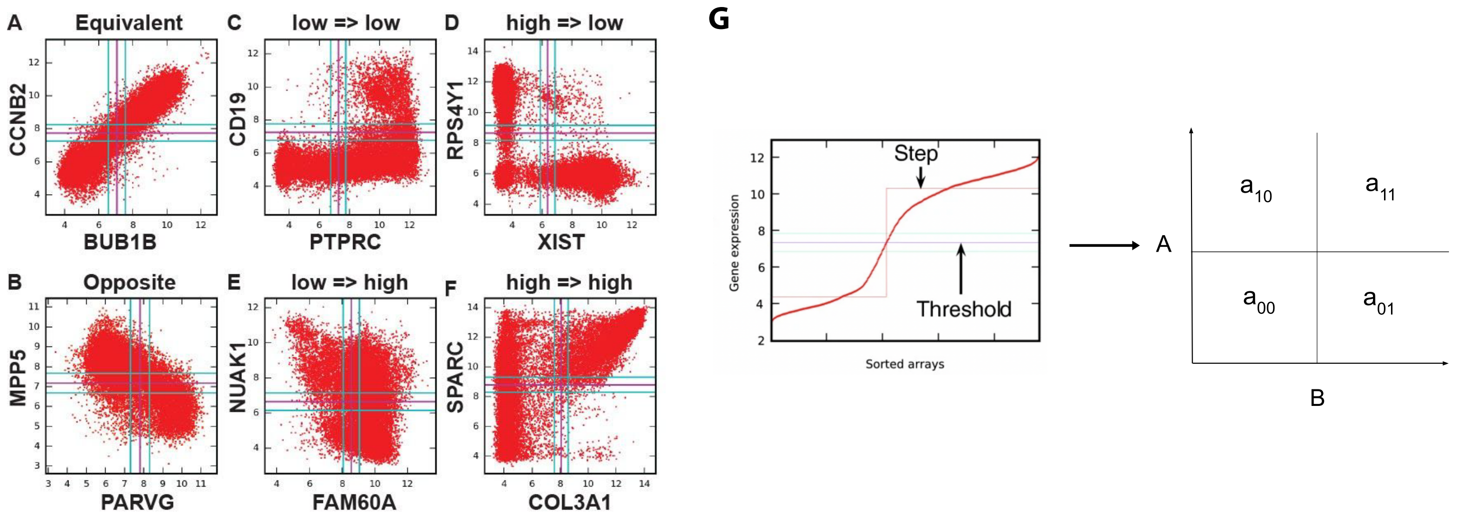

H

$$nA_{low} = (a_{00} + a_{01})$$
$$nB_{low} = (a_{00} + a_{10})$$
$$\text{total} = a_{00} + a_{01} + a_{10} + a_{11}$$
$$\hat{e} = \frac{\frac{nA_{low}}{\text{total}} \cdot \frac{nB_{low}}{\text{total}}}{\text{total}}$$
$$S_{00} = \frac{\hat{e} - n}{\sqrt{\hat{e}}}$$
$$p_{00} = \frac{1}{2}\left(\frac{a_{00}}{(a_{00} + a_{01})} + \frac{a_{00}}{(a_{00} + a_{10})}\right)$$
$$\text{Boolean Implication} = S > 2.5, p < 0.35$$

I

$$\text{Equivalent} = S_{01} > 2.5, p_{01} < 0.35, S_{10} > 2.5, p_{10} < 0.35$$
$$\text{Opposite} = S_{00} > 2.5, p_{00} < 0.35, S_{11} > 2.5, p_{11} < 0.35$$
$$A \text{ low} \implies B \text{ high} = (S_{00} > 2.5, p_{00} < 0.35)$$
$$A \text{ low} \implies B \text{ low} = (S_{01} > 2.5, p_{01} < 0.35)$$
$$A \text{ high} \implies B \text{ high} = (S_{10} > 2.5, p_{10} < 0.35)$$
$$A \text{ high} \implies B \text{ low} = (S_{11} > 2.5, p_{11} < 0.35)$$

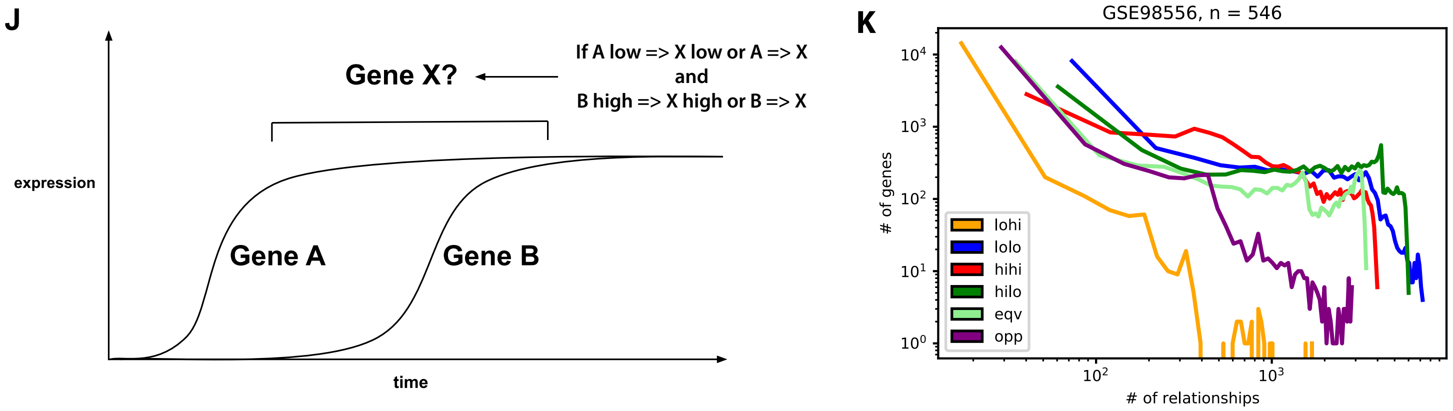

Figure S1. Method for discovering and applying Boolean implication relationships in single cell RNA sequencing data. (A-F): Six types of Boolean implication relationships are visible on scatter plots. Two are symmetric with two sparse quadrants (A,B) and four are asymmetric with one sparse quadrant (C-F). (G): This plot is divided into four quadrants based on thresholds identified by the StepMiner algorithm. (H-I): The BooleanNet algorithm identifies the sparse quadrants using a statistic S and a likelihood error rate p and applying thresholds of 2.5 and 0.35, respectively.
(J): Analysis of Boolean implication relationships was used to find genes involved in cell fate determination using bait genes (A and B). genes (A and B). (K): Distribution of the six types of Boolean implication relationship (as seen in A-F) in the single-cell dataset GSE98556. The log-log plot shows a histogram of the number of each relationship in GSE98556 and the number of genes exhibiting that relationship.

A: GSE148077

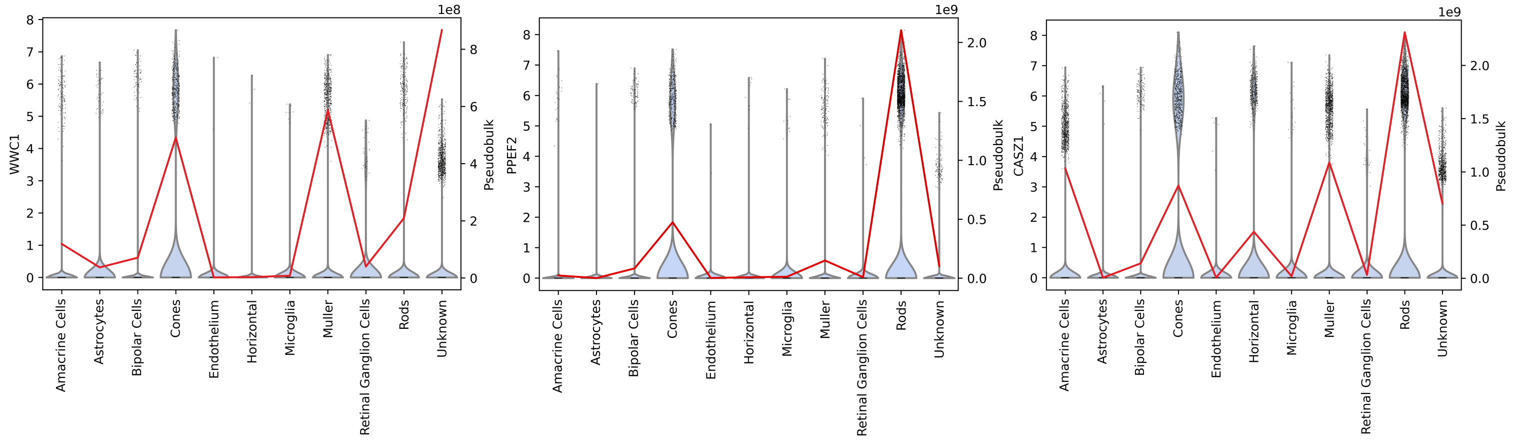

B: GSE138002

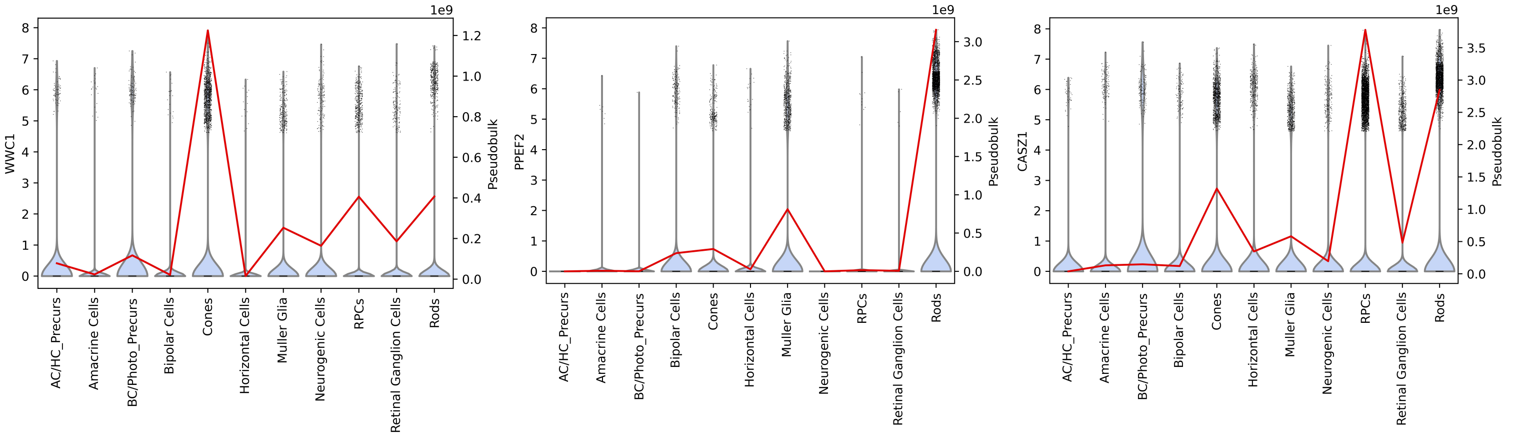

C: GSE122970

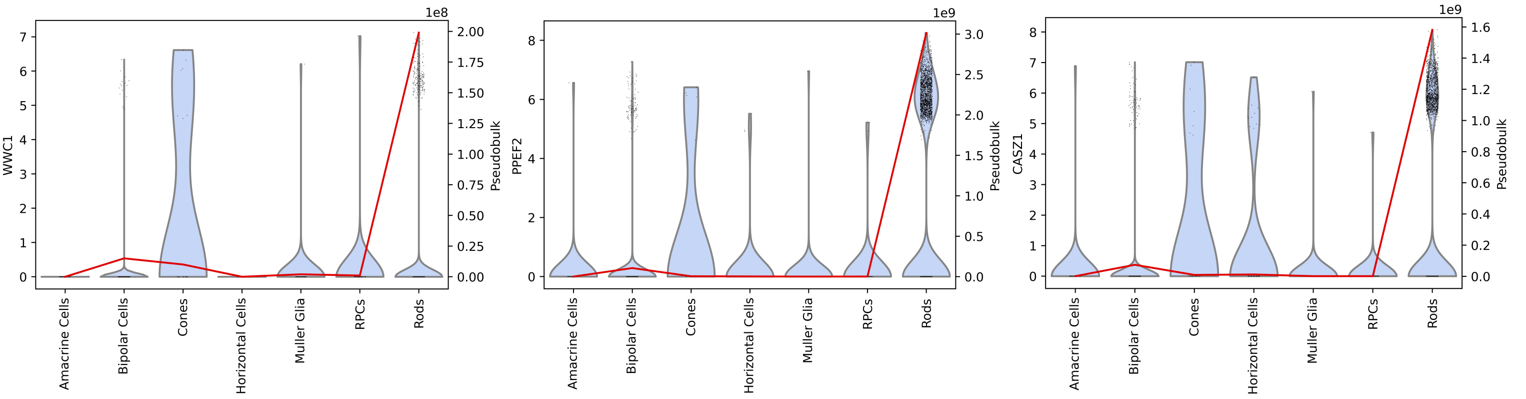

Supplementary Figure 2. (A): Violin plots in the Peng 2020 dataset (GSE148077). (B): Violin plots in adult retinal cells of the Lu 2020 dataset (GSE138002). (C): Violin plots in human neonatal retina of the Lu 2020 dataset (GSE122970). Violin plots (left x-axis) are generated from log-normalized CPM values. Line graphs (right x-axis) represent pseudobulk expression values for each cell type, normalized using CPM. WWC1 is a proposed cone photoreceptor gene, and CASZ1 and PPEF2 are proposed rod photoreceptor genes.
